# A Fixed Cohort Field Study of Gene Expression in Circulating Leukocytes From Dairy Cows With and Without Mastitis

**DOI:** 10.3389/fvets.2020.559279

**Published:** 2020-09-30

**Authors:** Craig S. McConnel, Sierra A. Crisp, Tyler D. Biggs, Stephen P. Ficklin, Lindsay M. Parrish, Sophie C. Trombetta, William M. Sischo, Amber Adams-Progar

**Affiliations:** ^1^Department of Veterinary Clinical Sciences, College of Veterinary Medicine, Washington State University, Pullman, WA, United States; ^2^Department of Horticulture, College of Agriculture, Human, and Natural Resource Sciences, Washington State University, Pullman, WA, United States; ^3^Department of Animal Sciences, College of Agriculture, Human, and Natural Resource Sciences, Washington State University, Pullman, WA, United States

**Keywords:** dairy, gene expression, innate immunity, leukocyte, mastitis

## Abstract

Specifically designed gene expression studies can be used to prioritize candidate genes and identify novel biomarkers affecting resilience against mastitis and other diseases in dairy cattle. The primary goal of this study was to assess whether specific peripheral leukocyte genes expressed differentially in a previous study of dairy cattle with postpartum disease, also would be expressed differentially in peripheral leukocytes from a diverse set of different dairy cattle with moderate to severe clinical mastitis. Four genes were selected for this study due to their differential expression in a previous transcriptomic analysis of circulating leukocytes from dairy cows with and without evidence of early postpartum disease. An additional 15 genes were included based on their cellular, immunologic, and inflammatory functions associated with resistance and tolerance to mastitis. This fixed cohort study was conducted on a conventional dairy in Washington state. Cows >50 days in milk (DIM) with mastitis (*n* = 12) were enrolled along with healthy cows (*n* = 8) selected to match the DIM and lactation numbers of mastitic cows. Blood was collected for a complete blood count (CBC), serum biochemistry, leukocyte isolation, and RNA extraction on the day of enrollment and twice more at 6 to 8-days intervals. Latent class analysis was performed to discriminate healthy vs. mastitic cows and to describe disease resolution. RNA samples were processed by the Primate Diagnostic Services Laboratory (University of Washington, Seattle, WA). Gene expression analysis was performed using the Nanostring System (Nanostring Technologies, Seattle, Washington, USA). Of the four genes (*C5AR1, CATHL6, LCN2*, and *PGLYRP1*) with evidence of upregulation in cows with mastitis, three of those genes (*CATHL6, LCN2*, and *PGLYRP1*) were investigated due to their previously identified association with postpartum disease. These genes are responsible for immunomodulatory molecules that selectively enhance or alter host innate immune defense mechanisms and modulate pathogen-induced inflammatory responses. Although further research is warranted to explain their functional mechanisms and bioactivity in cattle, our findings suggest that these conserved elements of innate immunity have the potential to bridge disease states and target tissues in diverse dairy populations.

## Introduction

Mastitis is one of the most economically impactful diseases of livestock (1). The incidence, severity, and outcomes associated with mastitis are influenced by several factors including pathogen types, environmental conditions, and host immunity (2). The immune response is a very complex biological process that must recognize the foreign organism, recruit immune cells, eliminate the invading pathogen, and resolve the inflammatory response. The speed, strength, and duration of this response are critically tied to the genetic background of an animal (3, 4), and genetic improvement offers the potential for cumulative, permanent and cost-effective disease resistance (5).

Genome-wide association studies (GWAS) are widely used to find DNA variants associated with complex traits such as mastitis (5–7). However, concordance among such studies is low and candidate genes for genetic selection are difficult to identify due to the complex interactions that must occur for effective disease resistance (5, 7). As an alternative, specifically designed gene expression studies in target tissues from healthy and affected animals can be used as biological evidence post GWAS to prioritize candidate genes and identify novel biomarkers (8). As such, transcriptomic profiling has identified genes, pathways, and regulatory networks activated during infection by various mastitis pathogens such as *Escherichia coli, Staphylococcus aureus*, and *Streptococcus uberis* (4).

Resilience against mastitis is influenced by many genes involved in multiple processes, including the response to infection, inflammation, and post-infection healing (7). For example, mammary gland associated candidate genes such as *IL1*β, *IL6, IL8, SAA3, TNF, IFNG, CD14, LBP, TLR2, TLR4*, and *C5AR1* (6) are responsible for a wide variety of cellular, immunologic, and inflammatory functions (9). More recently, Chen et al. combined GWAS and differential gene expression (DGE) data analyses to identify candidate genes expressed differentially in mammary tissue samples from cows specifically affected with *E. coli* and *S. uberis* mastitis (5). Ingenuity Pathway Analysis (IPA) highlighted signaling pathways comprising a network regulating the activity of leukocytes, especially neutrophils, during mammary gland inflammation. This suggested that polymorphisms in key genes in these pathways such as *ACTR3, BCAR1, CXCL2, CXCL6, CXCL8, FABP, SELP*, and *SELL* may influence dairy cow resilience against mastitis. Similarly, Cai et al. used Gene Ontology (GO) terms, Kyoto Encyclopedia of Genes and Genomes (KEGG) pathway analysis, and the mammalian phenotype database post GWAS to highlight putative causal genes affecting resistance to clinical mastitis (7). Results were confirmed using gene expression data from *E. coli*-challenged cow udders and highlighted genes with putative functions such as “abnormal humoral immune response,” “inflammatory response,” “innate immune response,” and “abnormal T cell physiology and decreased T cell proliferation.” These results indicate that the timely and properly controlled movement of leukocytes to infection loci may be key to an animal's resilience based on balancing pathogen elimination (resistance) and tissue damage (tolerance) (5, 10, 11).

Although resistance mechanisms are viewed as the primary function of immunity, disease tolerance also is essential in terms of tissue damage control mechanisms that limit the health and fitness costs of infection (12). Ultimately, resistance and tolerance to infection are integrated through stress and damage responses that regulate how tissues respond to cytokines and other cues emanating from immune cells. Given the variability in homeorhetic responses to diverse diseases, identifying common, conserved resistance and tolerance mechanisms should provide insight into resilience in the face of pathogenic microbes from different sites of infection. For example, altered expression profiles of peripheral leukocyte immune regulatory genes related to trafficking, migration, adhesion, energy metabolism, inflammatory mediators, cytokines, cell survival and apoptosis have been associated with both mastitis and uterine disease (13–17). These findings suggest that changes in peripheral leukocyte gene expression may emphasize mechanisms of immunity that bridge disease states and target tissues.

We recently identified 15 genes differentially expressed in peripheral blood leukocytes from dairy cattle with postpartum disease including clinical metritis, co-morbidities, and associated physiological changes (unpublished data). Although most of these 15 genes remain largely unreviewed in cattle, cross-species analysis suggests that they are primarily involved in immune cell and receptor function, tissue repair, and cell signaling based on functional enrichment analysis and gene ontology annotations (9). Four of the genes (*CATHL6, IL17D, LCN2*, and *PGLYRP1*) have useful background information related to underlying immunologic functions. Three of these genes (*CATHL6, LCN2*, and *PGLYRP1*) have been associated previously with mastitic somatic cells or mammary epithelial cells and their affiliated peptides play a role in disease resistance through potent antimicrobial activity (18–20). The fourth gene (*IL17D*) has not been extensively investigated but appears to play a role in both disease resistance and tolerance through the regulation of cytokine production involved in the inflammatory response (21). Of the 15 differentially expressed genes in our postpartum study, *PGLYRP1* was notable as a marker for diverse postpartum disease. Based on these data, the primary goal of this study was to assess whether specific peripheral leukocyte genes expressed differentially in dairy cattle with postpartum disease, also would be expressed differentially in peripheral leukocytes from a diverse set of cows with clinical mastitis. Specifically, we hypothesized that the top ranked gene (*PGLYRP1*) within our postpartum study would be expressed differentially in peripheral leukocytes from dairy cows of different breeds, parities, days in milk, production levels and pregnancy status with and without moderate to severe mastitis (grade 2 or 3) during the post-voluntary waiting period (>50 DIM).

## Materials and Methods

### Experimental Design and Clinical Assessments

This fixed cohort field study was conducted from February to April 2019, on a conventional dairy in the state of Washington with an inventory of ~4,000 lactating cows and an 8,464 kg average 305ME. Of note, the participating dairy was separate in terms of ownership and cows from the dairy that participated in our previous study of postpartum disease. All cows were housed in open dry lots or freestall barns, fed a total mixed ration, and milked 2 or 3 times daily. Eligible cows were >50 days in milk (DIM), with no recorded episodes of mastitis within the previous 30 days. Cows identified by farm personnel with mastitis were evaluated by a Washington State University veterinarian on the day of initial diagnosis and only cows with moderate to severe mastitis (grade 2 or 3) in a single mammary gland quarter were enrolled. Mastitis severity was based on abnormal milk (California Mastitis Test (CMT) ≥1; ≥400,000 SCC) and redness or swelling of the mammary gland without (grade 2) or with (grade 3) pyrexia, but no other clinical signs of systemic illness such as depression, anorexia, or dehydration (22). Healthy cows without mastitis or any other clinical disease were enrolled to provide comparisons to those cows with mastitis based on similar DIM, lactation number, and pregnancy status. All enrolled cows were evaluated cow-side on the day of enrollment and twice more by the WSU veterinarian at 6–8-days intervals to ascertain the progression in mammary gland condition, CMT score, rectal temperature, and β-hydroxybutyrate levels over a period of 14–15 days. Any treatments for mastitis were administered after initial sampling and at the discretion of the farm manager according to on-farm protocols.

Requirements for sample size were based on expectations for a multivariate analysis estimating the log fold-change in *PGLYRP1* DGE following a negative binomial distribution and an assay dynamic range of 100–200,000 counts for any given gene (nSolver Analysis Software 4.0, NanoString Technologies Inc., Seattle WA) (23). This equated to an estimate of a minimum of 8 animals within each group (mastitis, healthy cohort) based on a significance level of 0.05, power of 80%, and expectation that no more than 10% of healthy cows and no fewer than 80% of cows with mastitis would demonstrate a conservative 2 fold estimate of effect size.

### Blood Sampling

At the time of enrollment and the two subsequent clinical evaluations, blood was collected from the coccygeal blood vessels for leukocyte isolation (~10 mL/cow), complete blood counts (CBC) (~10 mL/cow), haptoglobin measurement (~10 mL/cow), and a serum biochemistry panel (~10 mL/cow). Whole blood for leukocyte isolation was collected into Tempus Blood RNA Tubes (Thermo Fisher, Waltham, MA). Blood for CBC evaluation was collected in Covidien Monoject™ coated EDTA evacuated tubes with lavender stoppers (Fisher Scientific, Waltham MA). Blood for haptoglobin and serum biochemistry analysis was collected in Covidien Monoject™ silicone-coated evacuated tubes with red stoppers (Fisher Scientific, Waltham MA). All tubes were inverted carefully multiple times and placed immediately on top of ice until further processing. Samples for CBC and biochemistry were kept chilled until submission to the WSU College of Veterinary Medicine clinical pathology laboratory within 6 h of collection.

### Haptoglobin

Blood to be analyzed for haptoglobin concentrations was processed within 6 h of collection at which point it was allowed to settle at room temperature for 30 min prior to centrifugation at 2,750 × g for 10 min. Separated serum was aspirated off the clot, placed into two 2 mL microcentrifuge tubes, and held at −80°C until further processing. Haptoglobin levels were obtained following instructions for the Bovine Haptoglobin ELISA Kit (Innovative Research, Inc.). Serum samples were initially diluted 1/500. Concentrations above the standard curve required additional dilutions of up to 1/50,000.

### Latent Class Analysis

Udder palpation, milk appearance, and CMT results provided a clinical diagnosis of mastitis. CBC and biochemistry data provided additional insight used to discriminate potentially healthy controls and gradients of mastitis severity. Latent class analysis (LCA) was performed using R (R Project for Statistical Computing, https://www.r-project.org/) and package poLCA to identify unique classes of animals with shared clinical and physiologic parameters based on categories for normal or abnormal using reference intervals established at the WSU clinical pathology laboratory (CBC and serum biochemistry) and Kansas State University veterinary diagnostic laboratory (haptoglobin). All observations from enrolled cows were used (*n* = 60 observations) and variables used to inform the models included: rectal temperature, β-hydroxybutyrate levels, serum haptoglobin levels, CBC values, and serum biochemistry values. LCA is a statistical method that uses observed categorical responses to identify underlying latent or “unobserved” groups of individuals or objects that share certain characteristics (24).

### Gene Selection

Nineteen candidate genes were selected for this study. Four genes (*CATHL6, IL17D, LCN2*, and *PGLYRP1*) were based on our previous transcriptomic analysis of circulating leukocytes in early postpartum dairy cows with postpartum disease and associated physiological changes (unpublished data). An additional 15 genes (*BCAR1, C5AR1, CD14, CXCL2, CXCL6, IFN6, IL1*β, *IL8, SAA3, SELL, STAT5A, TLR2, TLR4*, and *TNF*) were selected based on their potential for expression in peripheral leukocytes and previously described cellular, immunologic, and inflammatory functions associated with resistance and tolerance to mastitis (5–7). Five genes were used for internal control [golgin subfamily A, member 5 (*GOLGA5*), oxysterol-binding protein-like 2 (*OSBPL2*), single-strand-selective monofunctional uracil-DNA glycosylase 1 (*SMUG1*), 14-3-3 protein zeta/delta (*YWHAZ*), and Actin, cytoplasmic 1 (Beta-actin) (*ACTB*)] based on their previous identification as suitable controls for peripheral leukocytes (25–28).

### Leukocyte RNA Extraction and Gene Expression Analysis

Extraction of RNA from peripheral blood leukocytes was accomplished following the Tempus Spin RNA isolation kit (Thermo Fisher, Waltham, MA). Three samples were selected at random to test RNA integrity with the Qubit RNA IQ Assay Kit using the Qubit Fluometer 4.0 (Invitrogen Carlsbad, CA). Sample quantity was also measured using the Qubit RNA BR Assay Kit. Prior to submission, RNA was diluted to a working concentration of ≈20 ng/μl, except for 4 samples with <20 ng/ul (range 1–11 ng/ul). RNA samples were processed by the Primate Diagnostic Services Laboratory (University of Washington, Seattle, WA). Gene expression analysis of the 19 target genes and five housekeeping genes was performed using the Nanostring System (Nanostring Technologies, Seattle, Washington, USA), as previously described (29). Gene expression was measured using a custom CodeSet for the selected genes in the Nanostring nCounter Analysis System (NanoString Technologies, Seattle, WA). The NanoString technology uses a digital color-coded barcode tag with single-molecule imaging that can detect and count hundreds of unique transcripts per reaction. The analysis of data was performed using nSolver Analysis Software 4.0, including nCounter Advanced Analysis (version 2.0, NanoString Technologies, Seattle, WA). Low count data was omitted from a given analysis by removing probes that fell below a standard threshold count value of 20. The nSolver software detects probes based on a doubling of the counts relative to the median count value of the negative control. More specifically, fold changes and p-values were calculated using nCounter default settings and a Benjamini-Yekutieli (B-Y) correction for multiple comparisons. The B-Y correction makes the assumption that there may be some biological connection between genes and returns moderately conservative estimates of false discovery rate (FDR). The FDR is the proportion of genes with equal or greater evidence for differential expression (i.e., equal or lower raw *p*-value) than are expected to be “false discoveries” due to chance.

## Results

### Enrollments and Clinical Assessments

Twenty Holstein-Friesian and Holstein-Friesian x Jersey cross lactating cows were enrolled in this project. Healthy cows (*n* = 8) were selected to match the DIM and lactation numbers of mastitic cows (*n* = 12; Table 1). At enrollment, the cattle were ≥50 DIM (range 72–282 DIM) and in their first through fifth parities. Monthly Dairy Herd Improvement Association milk production test results were available demonstrating an average production level per cow at enrollment of 55.6 lb (25.2 kg) with a range of 31.0–97.0 lb (14.1–44.0 kg). Fifteen of the cows were pregnant at the time of enrollment with gestation length ranging from 117 to 200 days (Table 1). Of note, this study purposely investigated a range of breeds, parities, days in milk, production, and pregnancies in an effort to explore peripheral leukocyte gene expression signals in a diverse community of animals in field conditions. Variations in udder, milk, and CMT characteristics highlighted the clinical variability of symptoms inherent to mastitis grades (Table 2). At the farm manager's discretion, two of the mastitic cows with grade 2 mastitis were left untreated and the remaining 10 mastitic cows were treated once daily with intramammary cephapirin sodium (ToDay, Boehringer Ingelheim Vetmedica, Inc.) for 2–3 days commencing on the day of enrollment. None of the eight cows without mastitis had treatments administered during the sampling period.

**Table 1:** Animal demographics at the time of enrollment including mastitis status, breed, parity, freshening date, days in milk, previous milk test results, pregnancy status, and days carrying calf if applicable.

**Cow ID**	**Mastitis (Y/N)**	**Breed^a^**	**Parity**	**Fresh date**	**DIM^b^**	**Prev milk test date**	**Prev milk test lb (kg)**	**Preg**	**DCC^c^**
3731	N	H	4	6/22/18	241	2/6/19	97 (44)	Y	150
5307	N	X	4	5/13/18	270	2/6/19	37 (17)	Y	195
5362	N	X	4	10/12/18	144	2/6/19	84 (38)	N	na
6081	N	X	3	5/27/18	282	3/5/19	71 (32)	Y	175
6246	N	X	3	7/21/18	212	2/18/19	48 (22)	Y	149
6748	N	X	3	6/24/18	260	3/11/19	31 (14)	Y	149
8494	N	X	2	9/18/18	182	3/19/19	41 (19)	Y	117
11150	N	X	1	12/8/18	87	3/5/19	51 (23)	N	na
3955	Y	H	5	10/16/18	146	3/6/19	81 (37)	N	na
4451	Y	H	4	5/16/18	267	2/6/19	43 (20)	Y	200
5291	Y	X	4	6/23/18	236	2/6/19	71 (32)	Y	147
5410	Y	X	3	5/25/18	269	2/18/19	63 (29)	Y	171
5754	Y	X	4	10/25/18	123	2/25/19	71 (32)	N	na
6341	Y	X	3	6/15/18	269	3/11/19	33 (15)	Y	135
6493	Y	H	3	6/16/18	254	2/25/19	63 (29)	Y	174
6645	Y	X	3	7/10/18	219	2/14/19	35 (16)	Y	154
6805	Y	X	3	7/29/18	200	2/14/19	47 (21)	Y	138
9028	Y	X	2	8/17/18	214	3/19/19	50 (23)	Y	121
11151	Y	X	1	12/15/18	72	2/25/19	47 (21)	N	na
20008	Y	H	4	5/30/18	253	2/7/19	48 (22)	Y	167

a *Breed: H, Holstein-Friesian; X, Holstein-Friesian x Jersey*.

b *DIM: days in milk*.

c *DCC: days carrying calf (na = not applicable)*.

**Table 2:** Mammary quarter affected by mastitis if applicable, and udder palpation, milk appearance, milk CMT, and mastitis grade at the time of enrollment.

**Cow ID**	**Mastitis (Y/N)**	**Affected quarter^a^**	**Udder palpation**	**Milk appearance**	**CMT^b^**	**Mastitis grade^c^**
3731	N	na	Soft, supple	Grossly normal	N	na
5307	N	na	Soft, supple	Grossly normal	N	na
5362	N	na	Soft, full	Grossly normal	N	na
6081	N	na	Soft, full	Grossly normal	N	na
6246	N	na	Soft, supple	Grossly normal	N	na
6748	N	na	Soft, full	Grossly normal	N	na
8494	N	na	Soft, full	Grossly normal	N	na
11150	N	na	Soft, full	Grossly normal	N	na
3955	Y	LR	Hard, swollen, hot	Serous	1	2
4451	Y	LR	Firm, swollen	Serous	2	2
5291	Y	LR	Firm, swollen, hot	Bloody, clots	3	2
5410	Y	RR	Firm, swollen	Clots	3	3
5754	Y	LF	Firm	Clots	3	2
6341	Y	LF	Firm, swollen, hot	Serosanguinous, clots	2	2
6493	Y	RR	Swollen	Grossly normal	1	2
6645	Y	LR	Firm	Grossly normal	2	2
6805	Y	LF	Firm, swollen	Serous	3	2
9028	Y	RF	Firm, swollen	Clots	3	2
11151	Y	LR	Firm, swollen	Clots	3	2
20008	Y	LR	Hard, swollen, hot	Clots	3	3

a *Affected quarter: na = not applicable; LR = left rear; RR = right rear; LF = left front; RF = right front*.

b *CMT, California mastitis test; N, negative; T, trace; 1, weak positive; 2, distinct positive; 3, strong positive*.

c *Mastitis grade: na, not applicable; 2, abnormal milk, redness or swelling of the mammary gland, and no pyrexia; 3 = abnormal milk, redness or swelling of the mammary gland, and pyrexia*.

Results are presented in Table 3 from the repeated clinical assessments of CMT, rectal temperature, β-hydroxybutyrate, and haptoglobin. Additional results related to CBC and serum biochemistry are presented in Supplemental Tables S1, S2, respectively. None of the enrolled cows displayed clinical signs of systemic illness such as depression, anorexia, or dehydration at any of the assessments. Two mastitic cows were pyrexic (>39.2°C) and two healthy cows were subclinically ketotic (β-hydroxybutyrate (≥1.2 and <3.0 mmol/L) at the time of enrollment (Table 3). One mastitic cow demonstrated subclinical ketosis at the third sampling point. None of the healthy cows exhibited excessive levels of inflammatory proteins; however, 10 of the mastitic cows had haptoglobin levels at enrollment above the reference interval, and 2 of those cows had persistently high levels at the second sampling point as well. The two mastitic cows with normal haptoglobin levels at enrollment were those that were left untreated.

**Table 3:** Diagnostic results for cows with and without mastitis at all sampling points including California mastitis test, rectal temperature, serum β-hydroxybutyrate, serum haptoglobin levels, and latent classes.

**Cow ID**	**Mastitis (Y/N)**	**Affected quarter^a^**	**Sample date**	**DIM^b^**	**CMT^c^**	**Rectal temp (<39.2^**°**^C)**	**β-HOB^d^ (<1.2 mmol/L)**	**Hapt^e^ (≤140 μg/mL)**	**Latent class (1-2)**
3731	N	na	2/18/19	241	N	38.4	0.4	14.1	1
			2/25/19	248	N	38.3	0.7	13.0	1
			3/5/19	256	N	38.1	0.6	14.0	1
5307	N	na	2/7/19	270	N	38.1	1.5	14.2	1
			2/14/19	277	N	38.4	0.8	11.5	1
			2/21/19	284	na	37.9	0.5	11.0	1
5362	N	na	3/5/19	144	N	38.1	0.2	17.1	1
			3/11/19	150	N	38.4	0.6	12.6	1
			3/19/19	158	N	38.4	0.8	11.6	1
6081	N	na	3/5/19	282	N	38.3	0.8	25.8	1
			3/11/19	288	N	38.6	0.9	14.0	1
			3/19/19	296	N	38.1	1.1	13.7	1
6246	N	na	2/18/19	212	N	38.2	0.3	13.0	1
			2/25/19	219	N	38.6	0.9	132.0	1
			3/5/19	227	N	38.2	0.4	14.9	1
6748	N	na	3/11/19	260	N	38.7	0.9	13.5	1
			3/19/19	268	N	38.6	1	46.5	1
			3/26/19	275	N	38.6	0.9	52.4	1
8494	N	na	3/19/19	182	N	38.5	1.4	15.1	1
			3/26/19	189	N	38.2	1.1	11.8	1
			4/2/19	196	N	38.2	0.8	13.2	1
11150	N	na	3/5/19	87	N	38.2	0.6	15.1	1
			3/11/19	93	N	37.9	0.9	13.6	1
			3/19/19	101	N	37.9	0.6	16.7	1
3955	Y	LR	3/11/19	146	1	38.1	0.6	3525.6	2
			3/19/19	154	T	38.4	0.5	12.4	2
			3/26/19	161	N	38.1	0.9	12.4	2
4451	Y	LR	2/7/19	267	2	37.8	0.8	635.4	2
			2/14/19	274	N	38.2	0.7	14.7	2
			2/21/19	281	na	38.2	0.4	14.2	1
5291	Y	LR	2/14/19	236	3	38.7	0.8	82.6	1
			2/21/19	243	1	38.2	0.8	12.2	1
			3/1/19	251	N	37.8	1.2	16.2	1
5410	Y	RR	2/18/19	269	3	39.4	0.1	2340.3	2
			2/25/19	276	1	37.9	0.8	76.3	2
			3/5/19	284	N	38.3	0.4	11.6	2
5754	Y	LF	2/25/19	123	3	39.0	0.8	1427.1	2
			3/5/19	131	1	37.9	0.8	11.8	2
			3/11/19	137	N	38.7	1	106.3	2
6341	Y	LF	3/11/19	269	2	38.1	0.8	2383.3	2
			3/19/19	277	2	38.4	0.7	13.1	1
			3/26/19	284	na	38.1	0.7	11.5	1
6493	Y	RR	2/25/19	254	1	37.8	0.7	12.1	2
			3/5/19	262	1	38.1	0.6	13.7	2
			3/11/19	268	1	38.6	1	36.5	2
6645	Y	LR	2/14/19	219	2	38.3	0.9	1055.6	2
			2/21/19	271	N	38.6	0.9	14.0	1
			3/1/19	271	N	37.8	0.7	11.1	1
6805	Y	LF	2/14/19	200	3	38.6	0.7	4107.6	2
			2/21/19	207	2	38.1	0.6	20.0	2
			3/1/19	215	1	38.1	0.7	17.5	2
9028	Y	RF	3/19/19	214	3	38.1	0.5	2375.6	2
			3/26/19	221	N	38.3	0.5	13.0	2
			4/2/19	228	N	38.1	0.7	13.4	2
11151	Y	LR	2/25/19	72	3	37.9	0.7	2739.6	2
			3/5/19	80	T	38.5	0.7	358.1	2
			3/11/19	86	T	38.1	1	70.5	1
20008	Y	LR	2/7/19	253	3	39.6	0.6	791.7	2
			2/14/19	260	1	38.3	0.5	146.9	2
			2/21/19	267	^*^	38.1	0.5	13.0	2

a *Affected quarter: na, not applicable; LR, left rear; RR, right rear; LF, left front; RF, right front*.

b *DIM, days in milk*.

c CMT (California mastitis test): na = dried off; N = negative; T = trace; 1 = weak positive; 2 = distinct positive; 3 = strong positive;

* *= missing data point*.

d *β-HOB: β-hydroxybutyrate*.

e *Hapt = haptoglobin*.

*Reference cut-points are provided within parentheses, and cells with gray coloration indicate values outside established reference ranges*.

Although individual healthy cows did demonstrate some mild shifts outside CBC and serum biochemical reference ranges, the overall clinical and diagnostic assessments indicated no evidence of mastitis or systemic physiological derangements that would exclude them from the healthy reference group. In contrast, CBC and serum biochemistry results for mastitic cows provided a nuanced perspective on the severity and progression of inflammatory and immunologic responses at the time of enrollment and across sampling points (Supplemental Tables S1, S2). In particular, white blood cell counts in general and band cell, segmented cell, and lymphocyte counts specifically, were frequently outside reference intervals. Similarly, levels of fibrinogen, albumin, and globulin were outside references intervals for many of the mastitic cows across multiple time points.

### Latent Class Analysis

LCA was used to help corroborate the separation between healthy cows vs. diseased cows, and to provide insight into the impact of physiological changes from one sampling period to the next. Models for up to six classes were explored and a 2-class model was used to distinguish unique groups based on the lowest Akaike information criterion (914.74) and parsimony (Table 3). Latent class 1 (LC1) membership (*n* = 33) typically described substantially normal or mild changes to physiologic, CBC, or biochemical parameters (Table 3, Supplemental Tables S1, S2). Latent class 2 (LC2; *n* = 27) typically indicated moderate to severe systemic inflammatory and immunologic changes. All eight of the healthy cows were designated to LC1 for all three sampling points. At enrollment, 11 of the mastitic cows were designated to LC2, and one mastitic cow was assigned to LC1. This cow remained in LC1 across all time points and consistently had normal values for inflammatory proteins, band cells, and segmented cells. However, she was retained in the mastitis group because at enrollment she had a swollen udder, serous discharge, strong positive CMT, and a 5-fold elevation in haptoglobin level (82.6 μg/mL) compared to the healthy cows (mean 16.0 ± 4.2 μg/mL). As the sampling periods progressed, four mastitic cows were reclassified from LC2 to LC1 as inflammatory and immunologic parameters returned to normal (Table 3). These results aligned with clinical perceptions that the mastitis was resolving. Overall, cow with mastitis demonstrated expected pathophysiologic gradients of disease severity and resolution indicative of variations in pathogens, treatments, and host resistance and tolerance mechanisms.

### Differential Gene Expression

**Figure 1:**
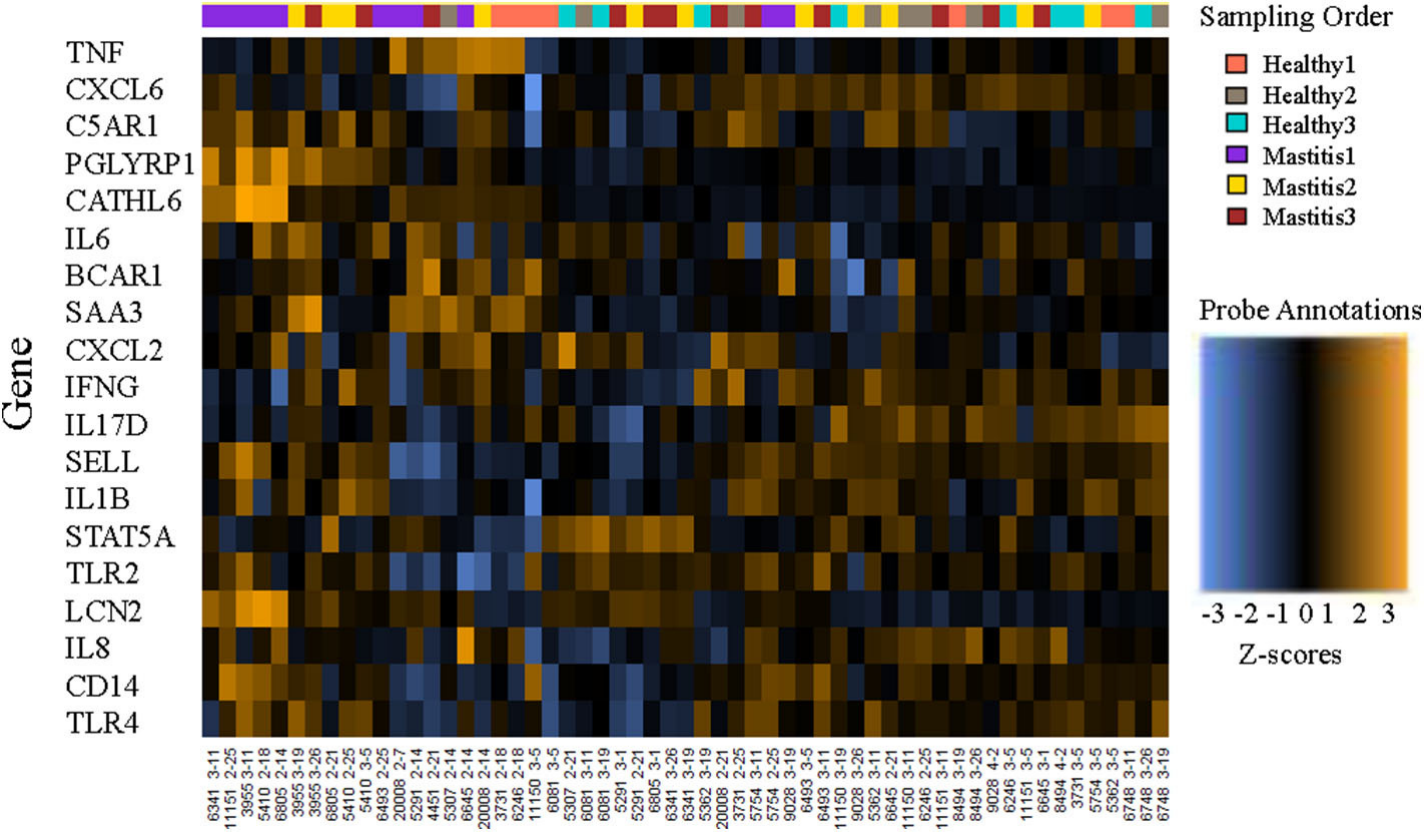
Heatmap of the normalized data, scaled to give all genes equal variance, generated via unsupervised clustering. Orange indicates high expression; blue indicates low expression. This plot is meant to provide a high-level exploratory view of the data.

RNA integrity number values ranged from 8.2 to 9.0 for the three randomly selected samples, indicating intact RNA and sufficient storage and extraction methods (30). Data from all 60 submissions passed QC, with no imaging, binding, positive control, or limit of detection flags. However, 3 of the samples with <20 ng/ul RNA (Cow ID 4451, 2/7/2019, 3 ng/ul; Cow ID 4451, 2/14/2019, 1 ng/ul; Cow ID 5307, 2/7/2019, 11 ng/ul) were flagged and removed from further analysis due to normalization factors well outside the recommended range (nSolver Analysis Software 4.0, NanoString Technologies, Seattle, WA). A heatmap of the remaining normalized data provides an overview of gene expression per cow at each sampling point (Figure 1).

There was no differential expression of genes between sampling points for healthy cows when comparing the initial samples to those from sampling points 2 or 3 (B-Y *p* > 0.8). Three genes (*PGLYRP1, CATHL6*, and *LCN2*) were differentially expressed between sampling points for mastitic cows when comparing samples from the onset of mastitis to those from sampling points 2 or 3 (B-Y *p* ≤ 0.05; Table 4). In confirmation of our hypothesis, *PGLYRP1* was expressed differentially in peripheral leukocytes from dairy cows with and without moderate to severe mastitis during the post-voluntary waiting period (B-Y *p* ≤ 0.05; Table 5). Two additional candidate genes chosen from our previous study of postpartum disease (*CATHL6* and *LCN2*) also were upregulated (B-Y *p* ≤ 0.05) at the onset of mastitis. When mastitic cows were compared to healthy cows by sampling point, *PGLYRP1* remained upregulated with notable log fold-changes from the onset of mastitis (4.31 ±1.55) through to sample 2 (2.15 ±1.06). On the other hand, the initial log fold-changes of *CATHL6* (4.70 ±1.70) and *LCN2* (3.75 ±1.26) quickly diminished such that they were upregulated only at the onset of mastitis. Based on the moderately conservative estimates of false discovery rate using the B-Y correction, none of the other candidate genes demonstrated DGE in association with mastitis (Table 5). It is worth acknowledging that a less conservative estimate (uncorrected *p* ≤ 0.05) indicated upregulation of one other candidate gene (*C5AR1*) at the onset of mastitis, and ongoing upregulation of *LCN2* and *PGLYRP1* at sampling points 2 and 3, respectively.

**Table 4:** Log fold-changes for peripheral leukocyte gene expression in cows with mastitis.

**Gene name**	**Mastitis sample**	**Log_**2**_ fold change**	**Std error (log_**2**_)**	**Lower CL (log_**2**_)**	**Upper CL (log_**2**_)**	***P*-value**	**B-Y *p*-value^a^**
*BCAR1*	2 vs. 1	−0.69	2.74	−6.06	4.68	0.8020	1.0000
	3 vs. 1	−0.18	2.67	−5.41	5.05	0.9470	1.0000
*C5AR1*	2 vs. 1	−0.02	0.28	−0.56	0.52	0.9420	1.0000
	3 vs. 1	−0.64	0.27	−1.17	−0.11	0.0241	0.2400
*CATHL6*	2 vs. 1	−5.11	0.64	−6.37	−3.85	0.0000	<0.0001
	3 vs. 1	−5.08	0.63	−6.31	−3.85	0.0000	<0.0001
*CD14*	2 vs. 1	−0.80	0.30	−1.39	−0.21	0.0127	0.1580
	3 vs. 1	−0.54	0.30	−1.12	0.03	0.0749	0.6210
*CXCL6*	2 vs. 1	−0.01	0.27	−0.54	0.52	0.9730	1.0000
	3 vs. 1	−0.06	0.26	−0.57	0.46	0.8350	1.0000
*IFNG*	2 vs. 1	0.79	1.14	−1.44	3.03	0.4930	1.0000
	3 vs. 1	0.60	1.11	−1.58	2.78	0.5960	1.0000
*IL1β*	2 vs. 1	0.42	0.26	−0.09	0.93	0.1160	0.9650
	3 vs. 1	−0.19	0.25	−0.68	0.31	0.4670	1.0000
*IL8*	2 vs. 1	−0.58	0.30	−1.16	0.00	0.0571	0.5690
	3 vs. 1	−0.73	0.29	−1.30	−0.16	0.0168	0.2090
*LCN2*	2 vs. 1	−2.67	0.53	−3.70	−1.64	0.0000	0.0004
	3 vs. 1	−2.60	0.52	−3.61	−1.59	0.0000	0.0005
*PGLYRP1*	2 vs. 1	−2.41	0.73	−3.84	−0.99	0.0023	0.0386
	3 vs. 1	−2.41	0.71	−3.80	−1.01	0.0020	0.0323
*SELL*	2 vs. 1	−0.27	0.30	−0.86	0.32	0.3740	1.0000
	3 vs. 1	−0.38	0.29	−0.96	0.19	0.1990	1.0000
*STAT5A*	2 vs. 1	0.11	0.09	−0.06	0.28	0.2240	1.0000
	3 vs. 1	0.08	0.09	−0.09	0.25	0.3870	1.0000
*TLR2*	2 vs. 1	−0.10	0.18	−0.45	0.26	0.5980	1.0000
	3 vs. 1	0.11	0.18	−0.23	0.46	0.5280	1.0000
*TLR4*	2 vs. 1	−0.14	0.21	−0.54	0.27	0.5150	1.0000
	3 vs. 1	−0.09	0.20	−0.48	0.31	0.6650	1.0000
*TNF*	2 vs. 1	−0.23	0.25	−0.72	0.26	0.3700	1.0000
	3 vs. 1	−0.31	0.25	−0.79	0.18	0.2210	1.0000

a *B-Y p-value: Benjamini-Yekutieli method for p-value adjustment*.

*Baseline samples at the onset of mastitis (n = 11), were compared to the second (n = 11) or third (n = 12) samples taken at 6 to 8-day intervals. Four genes (CXCL2, IL17D, IL6 and SAA3) were removed for falling below the background level too frequently*.

**Table 5:** Log fold-changes for peripheral leukocyte gene expression in cows with mastitis (*n*=12) vs. healthy cows (*n* = 8).

**Gene name**	**Sample**	**Log_**2**_ fold change**	**Std error (log_**2**_)**	**Lower CL (log_**2**_)**	**Upper CL (log_**2**_)**	***P-*value**	**B-Y *p*-value^a^**
*BCAR1*	1	−0.07	4.72	−9.32	9.19	0.9890	1.0000
	2	−0.54	3.34	−7.08	6.00	0.8730	1.0000
	3	0.39	3.37	−6.21	6.99	0.9100	1.0000
*C5AR1*	1	0.89	0.32	0.27	1.52	0.0138	0.1570
	2	0.09	0.33	−0.56	0.74	0.7930	1.0000
	3	0.20	0.28	−0.34	0.73	0.4880	1.0000
*CATHL6*	1	4.70	0.86	3.00	6.39	0.0001	0.0010
	2	0.07	0.94	−1.78	1.92	0.9420	1.0000
	3	0.89	1.38	−1.82	3.61	0.5270	1.0000
*CD14*	1	0.47	0.44	−0.39	1.32	0.3030	1.0000
	2	−0.33	0.21	−0.75	0.09	0.1380	1.0000
	3	0.00	0.22	−0.43	0.44	0.9840	1.0000
*CXCL2*	1	Removed					
	2	−0.09	0.90	−1.86	1.68	0.9240	1.0000
	3	−0.11	0.91	−1.89	1.67	0.9020	1.0000
*CXCL6*	1	0.36	0.38	−0.38	1.10	0.3550	1.0000
	2	−0.02	0.28	−0.57	0.53	0.9460	1.0000
	3	−0.13	0.27	−0.66	0.41	0.6510	1.0000
*IFNG*	1	Removed					
	2	−0.24	1.46	−3.10	2.63	0.8740	1.0000
	3	−0.36	1.97	−4.21	3.49	0.8560	1.0000
*IL17D*	1	Removed					
	2	−0.65	1.35	−3.30	2.01	0.6400	1.0000
	3	−0.72	1.14	−2.96	1.51	0.5340	1.0000
*IL1β*	1	0.44	0.38	−0.31	1.18	0.2710	1.0000
	2	0.34	0.23	−0.11	0.79	0.1570	1.0000
	3	−0.02	0.25	−0.51	0.47	0.9330	1.0000
*IL8*	1	0.71	0.44	−0.16	1.57	0.1320	1.0000
	2	−0.23	0.27	−0.76	0.30	0.4030	1.0000
	3	−0.22	0.35	−0.91	0.47	0.5420	1.0000
*LCN2*	1	3.75	0.65	2.49	5.01	<0.0001	0.0010
	2	0.97	0.43	0.14	1.81	0.0359	1.0000
	3	0.81	0.43	−0.03	1.66	0.0765	1.0000
*PGLYRP1*	1	4.31	0.79	2.76	5.86	0.0001	0.0010
	2	2.15	0.54	1.09	3.20	0.0009	0.0546
	3	1.88	0.63	0.65	3.10	0.0076	0.4460
*SELL*	1	0.66	0.37	−0.06	1.39	0.0925	0.8420
	2	0.00	0.24	−0.47	0.46	0.9850	1.0000
	3	−0.14	0.24	−0.60	0.33	0.5680	1.0000
*STAT5A*	1	0.13	0.09	−0.06	0.32	0.1900	1.0000
	2	0.03	0.11	−0.19	0.24	0.7910	1.0000
	3	−0.10	0.10	−0.29	0.09	0.3330	1.0000
*TLR2*	1	0.08	0.25	−0.41	0.57	0.7430	1.0000
	2	−0.23	0.13	−0.49	0.03	0.1010	1.0000
	3	−0.09	0.13	−0.33	0.16	0.5070	1.0000
*TLR4*	1	0.19	0.22	−0.24	0.63	0.4000	1.0000
	2	−0.17	0.22	−0.59	0.26	0.4570	1.0000
	3	−0.12	0.19	−0.50	0.25	0.5330	1.0000
*TNF*	1	−0.08	0.38	−0.82	0.66	0.8370	1.0000
	2	−0.04	0.25	−0.54	0.45	0.8680	1.0000
	3	−0.02	0.16	−0.33	0.28	0.8890	1.0000

a *B-Y p-value: Benjamini-Yekutieli method for p-value adjustment*.

*Comparisons were made based on sample number (e.g., initial mastitis samples were compared against initial healthy samples, etc.). Four genes were removed from specific analyses for falling below the background level too frequently either at the first sampling point (CXCL2, IL17D, IL6) or altogether (SAA3)*.

## Discussion

This study investigated differences in peripheral leukocyte gene expression in a diverse set of cows with and without moderate to severe mastitis and associated physiological changes and treatment. We identified three genes (*CATHL6, LCN2*, and *PGLYRP1*) that were upregulated during the early stages of mastitis regardless of differences in breeds, parities, days in milk, production levels and pregnancy status. These genes were investigated due to their previously identified association with diverse postpartum disease (unpublished data), and their role in disease resistance through the antimicrobial activity of their associated peptides (18, 31, 32). Our expectation was that if the four genes selected from the postpartum study went undetected in this study (e.g., *IL17D*), mammary-associated genes might serve as a DGE baseline for mastitis-associated immunologic and cellular stimuli. Ultimately, the disparity in DGE between the various candidate genes was likely the result of inherent influences related to pathogen diversity, variable tissue damage, and concomitant cytokines, chemokines and virulence factors. As pointed out previously, cellular effects may be diverse depending on the pathogens and the nature of inflammation which ultimately influences the expression of associated resistance and tolerance mechanisms. More specifically, it should be mentioned that although mRNA expression is a useful mechanism for investigating intracellular events in leukocytes, mRNA undergoes posttranscriptional modifications leading to certain limitations when studying innate immunity (33).

The cathelicidin-6 protein (*CATHL6* gene) is a member of a major group of host-defense antimicrobial peptides and has demonstrated potent antimicrobial activity against gram-negative and gram-positive bacteria, including methicillin-resistant *Staph. aureus* and *E. coli* (18, 34). Lipocalin-2 (*LCN2* gene) is also a member of a highly diverse group of proteins that participate in modeling the immune response (35). In certain conditions it may play a protective role and prevent severe tissue damage by facilitating tissue remodeling (36, 37). LCN2 rapidly increases during bacterial infections and inflammatory conditions (37), acts as a natural bacteriostatic agent through the interference with siderophore-mediated iron acquisition (31), and has been recommended as a potential marker of infection for bovine mastitis (20). Peptidoglycan recognition protein 1 (*PGLYRP1* gene) has been shown to have both gram-positive (*Staph. aureus* and *Listeria monocytogenes*) and gram-negative (*E. coli* and *Salmonella enterica* serovar Typhimurium) bactericidal effects (32, 38, 39). Furthermore, the *PGLYRP1* gene has been associated with variable disease resistance indicating potentially different roles for PGLYRP-1 in diverse diseases (19, 40). More broadly, peptidoglycan recognition proteins are important pattern recognition molecules of the innate immune system that have been shown to act like certain glycopeptide antibiotics (41, 42).

None of the other genes evaluated in this study demonstrated DGE associated with mastitis based on moderately conservative estimates of FDR. This included *IL17D* which was upregulated in our postpartum study but left undetected or inconsequential in this study. Although interleukin-17D (*IL17D* gene) has not been extensively investigated it belongs to the IL-17 family of cytokines which have been implicated in inflammation and host defense (21). However, the cellular sources of IL-17D are unknown and IL-17D-mediated cellular effects may be diverse depending on the pathogens and the nature of inflammation (43). Although our selection of other candidate genes was based on previously described associations with mastitis (5–7), their potential for expression in peripheral leukocytes was varied. Nonetheless, of the remaining candidates there were surprises within the omissions given their associated cellular, immunologic, and inflammatory functions underlying disease resistance and tolerance (9). The lack of DGE for *TLR4* was particularly surprising given the relationship between the C5AR1 and TLR4 signaling pathways (44), the role of TLR4 in mediating the innate immune response to bacterial lipopolysaccharide (9), and recent evidence for upregulation of TLR4 in peripheral blood leukocytes from cows with clinical mastitis (16).

A less conservative estimate of DGE within our study results suggested that *C5AR1* also might have been expressed differentially in mastitic cows. Although *C5AR1* was not identified as a candidate gene within our previous postpartum study, perhaps due to lactation stage effects (33), it was selected for this study due to its integrated resistance and tolerance mechanisms and association with mastitis traits in expression studies (6, 44). The C5a anaphylatoxin receptor (C5AR1) is specifically involved in the homeostatic response of bovine neutrophils to anaphylatoxin complement component 5a (C5a) (44). C5a is a member of the anaphylatoxins that represent endogenous danger signals that induce inflammatory responses and modulate innate immune cell functions, but also have major potential for harm to go along with their broad spectrum of biological functions (45, 46). The complexity of C5AR1-induced effects on different immune functions and systems is reflected by the fact that even though C5a–C5AR1 interaction is associated with the preservation of neutrophil innate immune functions (chemotaxis, phagocytosis, respiratory burst) and attenuation of the inflammatory reaction, excessive interaction of C5a–C5AR1 may result in harmful effects including the paralysis of neutrophil function during extreme inflammatory reactions such as sepsis (47, 48). Furthermore, there appears to be cross-talk between the C5AR1 and toll-like receptor 4 (TLR4) signaling pathways in neutrophils. This suggests that a better understanding of the attributes of C5AR1 and its interactions with C5a and the TLR4 pathways could prove useful particularly for maximizing dairy cow resilience against extreme disease outcomes such as sepsis (44).

The 4 genes (*C5AR1, CATHL6, LCN2*, and *PGLYRP1*) with evidence of DGE in this study encompass a range of antimicrobial and immunomodulatory activities, speaking to the importance of both disease resistance and tolerance in systemic resilience. Understanding this resilience is essential for managing the health of livestock and achieving a proper balance between pathogen elimination and excessive tissue damage (5, 49). Although the concept of resilience takes on different meanings depending on the context, it invariably relates to the ability of a system to maintain specific functions in the face of change (50). With reference to GO terms aligned with our findings, the specific molecular functions and biological processes influencing dairy cow resilience in the face of diverse diseases include the antimicrobial immune response mediated by antimicrobial peptides (51). In fact, host defense (antimicrobial) peptides such as CATHL6, LCN2, and PGLYRP-1 are attractive candidates for therapeutic development and represent potential alternatives to antimicrobials for infection management (52). They impact disease resistance through direct antimicrobial activities, and they influence disease tolerance through multifaceted immunomodulatory activities, including profound anti-infective and selective anti-inflammatory properties (53). These biological properties suggest that such host defense peptides and their synthetic derivatives likely possess wide-ranging clinical and diagnostic potential beyond antimicrobial replacement. Although further research is warranted to explain their functional mechanisms and bioactivity in cattle, our findings suggest that these conserved elements of innate immunity have the potential to bridge disease states and target tissues in diverse dairy populations.

## Data Availability Statement

All datasets presented in this study are included in the article/Supplementary Material.

## Ethics Statement

This animal study was reviewed and approved by Washington State University Institutional Animal Care and Use Committee ASAF#05061. Written informed consent was obtained from the owner for the participation of animals in this study.

## Author Contributions

CM, SC, WS, and AA-P contributed to the conception and design of the study. CM, SC, ST, and LP performed the field trial and laboratory work. TB, SF, and WS performed bioinformatics analysis. CM, SC, and WS wrote the first draft of the manuscript. All authors listed have made a substantial, direct and intellectual contribution to the work, and approved the submitted version.

## Conflict of Interest

The authors declare that the research was conducted in the absence of any commercial or financial relationships that could be construed as a potential conflict of interest.

## References

[1] Seegers H Fourichon C Beaudeau F. Production effects related to mastitis and mastitis economics in dairy cattle herds. Vet Res. (2003) 34:475–91. 10.1051/vetres:2003027 14556691

[2] De Vliegher S Fox LK Piepers S McDougall S Barkema HW. Invited review. Mastitis in dairy heifers. nature of the disease, potential impact, prevention, and control. J Dairy Sci. (2012) 95:1025–40. 10.3168/jds.2010-4074 22365187

[3] Pighetti GM Elliott AA. Gene polymorphisms. the keys for marker assisted selection and unraveling core regulatory pathways for mastitis resistance. J Mammary Gland Biol Neoplasia. (2011) 16:421–32. 10.1007/s10911-011-9238-9 21997401

[4] Rinaldi M Li RW Capuco AV. Mastitis associated transcriptomic disruptions in cattle. Vet Immunol Immunopathol. (2010) 138:267–79. 10.1016/j.vetimm.2010.10.005 21040982

[5] Chen X Cheng Z Zhang S Werling D Wathes DC Combining genome wide association studies and differential gene expression data analyses identifies candidate genes affecting mastitis caused by two different pathogens in the dairy cow. Open J Anim Sci. (2015) 5:358–93. 10.4236/ojas.2015.54040

[6] Ogorevc J Kunej T Razpet A Dovc P. Database of cattle candidate genes and genetic markers for milk production and mastitis. Anim Genet. (2009) 40:832–51. 10.1111/j.1365-2052.2009.01921.x 19508288 PMC2779988

[7] Cai Z Guldbrandtsen B Lund MS Sahana G. Prioritizing candidate genes post-GWAS using multiple sources of data for mastitis resistance in dairy cattle. BMC Genomics. (2018) 19:656. 10.1186/s12864-018-5050-x 30189836 PMC6127918

[8] Fang L Sahana G Su G Yu Y Zhang S Lund MS. Integrating sequence-based GWAS and RNA-Seq provides novel insights into the genetic basis of mastitis and milk production in dairy cattle. Sci Rep. (2017) 7:45560. 10.1038/srep45560 28358110 PMC5372096

[9] The UniProt Consortium. UniProt. a worldwide hub of protein knowledge. Nucleic Acids Res. (2018) 47:D506–15. 10.1093/nar/gky1049 30395287 PMC6323992

[10] Medzhitov R Schneider DS Soares MP. Disease tolerance as a defense strategy. Science. (2012) 335:936–41. 10.1126/science.1214935 22363001 PMC3564547

[11] Schneider DS Ayres JS. Two ways to survive infection. what resistance and tolerance can teach us about treating infectious diseases. Nat Rev Immunol. (2008) 8:889–95. 10.1038/nri2432 18927577 PMC4368196

[12] Martins R Carlos AR Braza F Thompson JA Bastos-Amador P Ramos S. Disease tolerance as an inherent component of immunity. Annu Rev Immunol. (2019) 37:405–37. 10.1146/annurev-immunol-042718-041739 30673535

[13] Düvel A Maass J Heppelmann M Hussen J Koy M Piechotta M. Peripheral blood leukocytes of cows with subclinical endometritis show an altered cellular composition and gene expression. Theriogenology. (2014) 81:906–17. 10.1016/j.theriogenology.2014.01.007 24560452

[14] Galvão KN Felippe MJB Brittin SB Sper R Fraga M Galvão JS Evaluation of cytokine expression by blood monocytes of lactating holstein cows with or without postpartum uterine disease. Theriogenology. (2012) 77:356–72. 10.1016/j.theriogenology.2011.08.008 21924475

[15] Bromfield JJ Watt MM Iacovides SM. Characterisation of peripheral blood mononuclear cell populations in periparturient dairy cows that develop metritis. Vet Immunol Immunopathol. (2018) 200:69–75. 10.1016/j.vetimm.2018.04.010 29776614 PMC6445263

[16] Yang F Chen F Li L Yan L Badri T Lv C. Three Novel Players. PTK2B, SYK, and TNFRSF21 were identified to be involved in the regulation of bovine mastitis susceptibility via gwas and post-transcriptional analysis. Front Immunol. (2019) 10:1579. 10.3389/fimmu.2019.01579 31447828 PMC6691815

[17] Tao W Mallard B. Differentially expressed genes associated with Staphylococcus aureus mastitis of Canadian Holstein cows. Vet Immunol Immunopathol. (2007) 120:201–11. 10.1016/j.vetimm.2007.06.019 17658619

[18] Tomasinsig L De Conti G Skerlavaj B Piccinini R Mazzilli M D'Este F. Broad-spectrum activity against bacterial mastitis pathogens and activation of mammary epithelial cells support a protective role of neutrophil cathelicidins in bovine mastitis. Infect Immun. (2010) 78:1781–8. 10.1128/IAI.01090-09 20100862 PMC2849419

[19] Wang HL Li ZX Wang LJ He H Yang J Chen L. Polymorphism in PGLYRP-1 gene by PCR-RFLP and its association with somatic cell score in Chinese Holstein. Res Vet Sci. (2013) 95:508–14. 10.1016/j.rvsc.2013.06.005 23820447

[20] Pokorska J Piestrzyńska-Kajtoch A Kułaj D Ochrem A Radko A Polymorphism of bovine lipocalin-2 gene and its impact on milk production traits and mastitis in Holstein Friesian cattle. Electron J Biotechn. (2019) 40:17–21. 10.1016/j.ejbt.2019.04.004

[21] Pappu R Ramirez-Carrozzi V Sambandam A. The interleukin-17 cytokine family. critical players in host defence and inflammatory diseases. Immunology. (2011) 134:8–16. 10.1111/j.1365-2567.2011.03465.x 21726218 PMC3173690

[22] Pinzon-Sanchez C Ruegg PL. Risk factors associated with short-term post-treatment outcomes of clinical mastitis. J Dairy Sci. (2011) 94:3397–410. 10.3168/jds.2010-3925 21700025

[23] Wang H Horbinski C Wu H Liu Y Sheng S Liu J. NanoStringDiff. A novel statistical method for differential expression analysis based on NanoString nCounter data. Nucleic Acids Res. (2016) 44:e151. 10.1093/nar/gkw677 27471031 PMC5175344

[24] Collins LM Lanza ST Latent Class and Latent Transition Analysis With Applications in the Social Behavioral, and Health Sciences. Hoboken, NJ: John Wiley and Sons, Inc (2010).

[25] Moyes KM Drackley JK Morin DE Loor JJ. Greater expression of TLR2, TLR4, and IL6 due to negative energy balance is associated with lower expression of HLA-DRA and HLA-A in bovine blood neutrophils after intramammary mastitis challenge with Streptococcus uberis. Funct Integr Genomics. (2010) 10:53–61. 10.1007/s10142-009-0154-7 20072847

[26] Spalenza V Girolami F Bevilacqua C Riondato F Rasero R Nebbia C. Identification of internal control genes for quantitative expression analysis by real-time PCR in bovine peripheral lymphocytes. Vet J. (2011) 189:278–83. 10.1016/j.tvjl.2010.11.017 21169039

[27] De Ketelaere A Goossens K Peelman L Burvenich C. Technical note. validation of internal control genes for gene expression analysis in bovine polymorphonuclear leukocytes. J Dairy Sci. (2006) 89:4066–9. 10.3168/jds.S0022-0302(06)72450-X 16960083

[28] Robinson TL Sutherland IA Sutherland J. Validation of candidate bovine reference genes for use with real-time PCR. Vet Immunol Immunopathol. (2007) 115:160–65. 10.1016/j.vetimm.2006.09.012 17074403

[29] Malkov VA Serikawa KA Balantac N Watters J Geiss G Mashadi-Hossein A. Multiplexed measurements of gene signatures in different analytes using the nanostring ncounter assay system. BMC Res Notes. (2009) 2:80. 10.1186/1756-0500-2-80 19426535 PMC2688518

[30] Schroeder A Mueller O Stocker S Salowsky R Leiber M Gassmann M. The RIN. an RNA integrity number for assigning integrity values to RNA measurements. BMC Mol Biol. (2006) 7:3. 10.1186/1471-2199-7-3 16448564 PMC1413964

[31] Goetz DH Holmes MA Borregaard N Bluhm ME Raymond KN Strong RK. The neutrophil lipocalin NGAL is a bacteriostatic agent that interferes with siderophore-mediated iron acquisition. Mol Cell. (2002) 10:1033–43. 10.1016/S1097-2765(02)00708-6 12453412

[32] Tydell CC Yuan J Tran P Selsted ME. Bovine peptidoglycan recognition protein-S. antimicrobial activity, localization, secretion, and binding properties. J Immunol. (2006) 176:1154–62. 10.4049/jimmunol.176.2.1154 16394004

[33] Stevens MG Peelman LJ De Spiegeleer B Pezeshki A Van De Walle GR Duchateau L. Differential gene expression of the toll-like receptor-4 cascade and neutrophil function in early- and mid-lactating dairy cows. J Dairy Sci. (2011) 94:1277–88. 10.3168/jds.2010-3563 21338793

[34] Whelehan CJ Barry-Reidy A Meade KG Eckersall PD Chapwanya A Narciandi F. Characterisation and expression profile of the bovine cathelicidin gene repertoire in mammary tissue. BMC Genomics. (2014) 15:128. 10.1186/1471-2164-15-128 24524771 PMC3932039

[35] Flower DR North ACT Sansom CE. The lipocalin protein family. structural and sequence overview. Biochim Biophys Acta. (2000) 1482:9–24. 10.1016/S0167-4838(00)00148-5 11058743

[36] Shashidharamurthy R Machiah D Aitken JD Putty K Srinivasan G Chassaing B. Differential role of lipocalin 2 during immune complex-mediated acute and chronic inflammation in mice. Arthritis Rheum. (2013) 65:1064–73. 10.1002/art.37840 23280250 PMC3618508

[37] Schmidt-Ott KM Mori K Li JY Kalandadze A Cohen DJ Devarajan P. Dual action of neutrophil gelatinase–associated lipocalin. J Am Soc Nephrol. (2007) 18:407–13. 10.1681/ASN.2006080882 17229907

[38] Tydell CC Yount N Tran D Yuan J Selsted ME. Isolation, characterization, and antimicrobial properties of bovine oligosaccharide-binding protein. A microbicidal granule protein of eosinophils and neutrophils. J Biol Chem. (2002) 277:19658–64. 10.1074/jbc.M200659200 11880375

[39] Wang M Liu LH Wang S Li X Lu X Gupta D. Human peptidoglycan recognition proteins require zinc to kill both gram-positive and gram-negative bacteria and are synergistic with antibacterial peptides. J Immunol. (2007) 178:3116–25. 10.4049/jimmunol.178.5.3116 17312159

[40] Pant SD Verschoor CP Schenkel FS You Q Kelton DF Karrow NA. Bovine PGLYRP1 polymorphisms and their association with resistance to mycobacterium avium ssp. paratuberculosis. Animal Genetics. (2011) 42:354–60. 10.1111/j.1365-2052.2010.02153.x 21749417

[41] Cho S Wang Q Swaminathan CP Hesek D Lee M Boons GJ. Structural insights into the bactericidal mechanism of human peptidoglycan recognition proteins. Proc Natl Acad Sci USA. (2007) 104:8761–6. 10.1073/pnas.0701453104 17502600 PMC1885576

[42] Kashyap DR Wang M Liu LH Boons GJ Gupta D Dziarski R. Peptidoglycan recognition proteins kill bacteria by activating protein-sensing two-component systems. Nat Med. (2011) 17:676–83. 10.1038/nm.2357 21602801 PMC3176504

[43] Lee Y Clinton J Yao C Chang SH. Interleukin-17D promotes pathogenicity during infection by suppressing CD8 T cell activity. Front Immunol. (2019) 10:1172. 10.3389/fimmu.2019.01172 31244826 PMC6562898

[44] Stevens MG Van Poucke M Peelman LJ Rainard P De Spiegeleer B Rogiers C. Anaphylatoxin C5a-induced toll-like receptor 4 signaling in bovine neutrophils. J Dairy Sci. (2011) 94:152–64. 10.3168/jds.2010-3358 21183027

[45] Riedemann NC Guo RF Ward PA. A key role of C5a/C5aR activation for the development of sepsis. J Leukoc Biol. (2003) 74:966–70. 10.1189/jlb.0403137 12960283

[46] Nemali S Siemsen DW Nelson LK Bunger PL Faulkner CL Rainard P. Molecular analysis of the bovine anaphylatoxin C5a receptor. J Leukocyte Biol. (2008) 84:537–49. 10.1189/jlb.0208142 18480166 PMC2493078

[47] Guo RF Riedemann NC Ward PA. Role of C5a-C5aR interaction in sepsis. Shock. (2004) 21:1–7. 10.1097/01.shk.0000105502.75189.5e 14676676

[48] Gerard C. Complement C5a in the sepsis syndrome–too much of a good thing? N Engl J Med. (2003) 348:167–9. 10.1056/NEJMcibr022995 12519929

[49] Scheffer M Bolhuis JE Borsboom D Buchman TG Gijzel SMW Goulson D. Quantifying resilience of humans and other animals. Proc Natl Acad Sci USA. (2018) 115:11883–11890. 10.1073/pnas.1810630115 30373844 PMC6255191

[50] Baggio JA Brown K Hellebrandt D Boundary object or bridging concept? A citation network analysis of resilience. Ecol Soc. (2015) 20:2 10.5751/ES-07484-200202

[51] Gaudet P Livstone MS Lewis SE Thomas PD. Phylogenetic-based propagation of functional annotations within the gene ontology consortium. Brief Bioinform. (2011) 12:449–62. 10.1093/bib/bbr042 21873635 PMC3178059

[52] Mookherjee N Hancock RE. Cationic host defence peptides. Innate immune regulatory peptides as a novel approach for treating infections. Cell Mol Life Sci. (2007) 64:922–33. 10.1007/s00018-007-6475-6 17310278 PMC11136131

[53] Hilchie AL Wuerth K Hancock RE. Immune modulation by multifaceted cationic host defense (antimicrobial) peptides. Nat Chem Biol. (2013) 9:761–8. 10.1038/nchembio.1393 24231617

