# Supplementary material for: A Fixed Cohort Field Study of Gene Expression in Circulating Leukocytes From Dairy Cows With and Without Mastitis
Source: Front Vet Sci. 2020 Sep 30;7:559279. doi: 10.3389/fvets.2020.559279 (PMC7554338; doi:10.3389/fvets.2020.559279)
Supplement: Supplementary file 1 [file Data_Sheet_1.pdf]

Table S1. Complete blood count data for cows with and without mastitis at all sampling points. Reference cut-points are provided within parentheses, and cells with grey coloration indicate values outside established reference ranges.

Cow ID	Mastitis (Y/N)	Sampling Dates	WBC[1] (5.5-13.5 $x10^3$/μL)	Band#[2] (≤0.1 $x10^3$/μL)	Seg#[3] (1.4-8.0 $x10^3$/μL)	Lymph#[4] (2.3-6.6 $x10^3$/μL)	Mono#[5] (≤1.4 $x10^3$/μL)	Eosin#[6] (≤0.8 $x10^3$/μL)	Baso#[7] (≤0.3 $x10^3$/μL)	Meta#[8] (0/μL)	RBC[9] (5.6-8.1 $x10^6$/μL)	Hgb[10] (9.5-12.6 g/dL)
3731	N	2/18/19	6.3	0.00	2.46	3.15	0.57	0.13	0.00	0.0	6.16	9.9
		2/25/19	6.3	0	3.402	2.205	0.32	0.38	0.00	0.0	6.25	10.3
		3/5/19	6.2	0.00	3.10	1.86	0.19	1.05	0.00	0.0	6.56	10.5
5307	N	2/7/19	9.2	0.00	2.21	6.26	0.37	0.37	0.00	0.0	6.15	9.3
		2/14/19	9.5	0.00	3.61	5.32	0.48	0.10	0.00	0.0	6.15	9.3
		2/21/19	7.3	0.00	2.70	3.94	0.29	0.37	0.00	0.0	5.71	8.9
5362	N	3/5/19	7.7	0.00	4.54	2.62	0.23	0.31	0.00	0.0	7.14	10.7
		3/11/19	6.4	0.00	3.90	2.18	0.13	0.13	0.06	0.0	7.3	10.7
		3/19/19	8.4	0.00	3.78	4.03	0.50	0.08	0.00	0.0	7.26	10.7
6081	N	3/5/19	11.7	0.00	6.44	5.15	0.12	0.00	0.00	0.0	6.91	11
		3/11/19	10.9	0.00	6.98	3.60	0.33	0.00	0.00	0.0	6.39	10.2
		3/19/19	12	0.00	3.72	7.80	0.48	0.00	0.00	0.0	6.77	11.1
6246	N	2/18/19	8.3	0.00	2.41	5.56	0.33	0.00	0.00	0.0	6.88	10.9
		2/25/19	9.7	0	3.589	5.432	0.58	0.10	0.00	0.0	6.64	10.5
		3/5/19	4.8	0.00	1.63	2.98	0.14	0.05	0.00	0.0	6.39	10.3
6748	N	3/11/19	6.5	0.00	3.12	3.06	0.20	0.07	0.07	0.0	6.96	9.8
		3/19/19	8.7	0.00	4.26	4.00	0.35	0.00	0.09	0.0	7.4	10.7
		3/26/19	9.3	0.00	4.84	3.91	0.37	0.09	0.09	0.0	7.28	10.5
8494	N	3/19/19	6.8	0.00	4.01	2.58	0.20	0.00	0.00	0.0	6.44	10.9
		3/26/19	6.2	0.00	3.10	2.54	0.25	0.19	0.12	0.0	6.2	10.7
		4/2/19	6.2	0.00	2.85	3.16	0.12	0.06	0.00	0.0	6.89	12.1
11150	N	3/5/19	6.1	0.00	3.17	2.38	0.24	0.31	0.00	0.0	6.32	10
		3/11/19	5.9	0.00	3.07	2.30	0.12	0.30	0.12	0.0	6.5	10.4
		3/19/19	6.1	0.00	2.44	3.23	0.18	0.24	0.00	0.0	6.32	10
3955	Y	3/11/19	11.4	2.51	5.36	1.37	0.34	1.37	0.23	0.2	5.47	9
		3/19/19	11.7	0.70	8.42	2.11	0.35	0.12	0.00	0.0	5.32	8.6
		3/26/19	8.2	0.00	6.40	1.56	0.16	0.08	0.00	0.0	5.28	8.6
4451	Y	2/7/19	14.9	0.75	2.68	11.03	0.45	0.00	0.00	0.0	6.83	10.4
		2/14/19	18.1	0.00	4.71	11.95	1.27	0.18	0.00	0.0	7.15	11.1

		2/21/19	18.5	0.00	5.55	12.21	0.37	0.19	0.00	0.0	6.93	10.5
		2/14/19	20.9	0.00	5.64	15.00	0.21	0.00	0.00	0.0	6.93	10
5291	Y	2/21/19	18.8	0.00	2.44	15.98	0.19	0.19	0.00	0.0	6.42	9.3
		3/1/19	20.9	0.00	2.09	16.72	1.25	0.84	0.00	0.0	7.04	10.4
		2/18/19	3.7	0.11	0.74	2.26	0.07	0.52	0.00	0.0	6.24	10
5410	Y	2/25/19	13.9	0.695	8.201	4.726	0.28	0.00	0.00	0.0	6.02	9.7
		3/5/19	13.9	0.14	8.48	4.31	0.83	0.14	0.00	0.0	5.91	9.7
		2/25/19	8.8	0.88	5.456	1.584	0.26	0.26	0.35	0.0	6.52	11.1
5754	Y	3/5/19	11.6	0.23	6.96	3.94	0.23	0.12	0.12	0.0	6.63	10.9
		3/11/19	9.3	0.00	5.30	3.72	0.28	0.00	0.00	0.0	6.34	10.5
		3/11/19	7.9	0.24	3.71	3.63	0.32	0.00	0.00	0.0	5.41	8.4
6341	Y	3/19/19	10.9	0.00	6.21	4.47	0.22	0.00	0.00	0.0	5.65	8.8
		3/26/19	10	0.00	3.80	5.50	0.20	0.40	0.10	0.0	6	9.4
		2/25/19	8.6	0.09	3.78	4.04	0.52	0.17	0.00	0.0	5.91	8.7
6493	Y	3/5/19	7.5	0.15	5.03	1.95	0.08	0.30	0.00	0.0	6.34	9.3
		3/11/19	5.2	0.00	3.43	1.72	0.05	0.00	0.00	0.0	5.52	8.3
		2/14/19	4.7	0.30	1.20	2.96	0.00	0.14	0.00	0.0	6.69	11.3
6645	Y	2/21/19	10.3	0.00	4.02	5.25	0.52	0.52	0.00	0.0	6.62	11
		3/1/19	9.5	0.00	4.85	3.80	0.38	0.38	0.10	0.0	6.9	11.5
		2/14/19	10.8	0.54	1.62	7.99	0.00	0.11	0.11	0.4	5.8	9.4
6805	Y	2/21/19	18.8	0.19	8.65	9.40	0.56	0.00	0.00	0.0	5.59	9.1
		3/1/19	19.8	0.00	4.75	14.26	0.20	0.59	0.00	0.0	5.52	8.8
		3/19/19	9.1	1.18	4.00	3.64	0.18	0.09	0.00	0.0	7.19	10.6
9028	Y	3/26/19	9.9	0.00	4.16	4.55	0.30	0.89	0.00	0.0	6.76	10.4
		4/2/19	7.8	0.00	3.35	3.59	0.39	0.39	0.08	0.0	6.79	10.1
		2/25/19	7.2	0.576	1.656	4.176	0.58	0.22	0.00	0.0	6.73	10.6
11151	Y	3/5/19	8.9	0.18	5.61	2.58	0.36	0.18	0.00	0.0	6.29	9.5
		3/11/19	7.5	0.00	3.75	3.53	0.23	0.00	0.00	0.0	6.15	9.3
		2/7/19	2.6	0.05	0.10	2.37	0.08	0.00	0.00	0.0	6.62	11.8
20008	Y	2/14/19	6.1	0.00	3.29	2.44	0.24	0.12	0.00	0.0	5.54	9.9
		2/21/19	6.7	0.00	3.22	2.75	0.27	0.47	0.00	0.0	5.6	10.2

PCV[11] (25-33%)	Pprot[12] (7.0-8.5 g/dL)	Fibrinogen (200-600 mg/dL)	MCV[13] (37-51 fL)	MCH[14] (14.5-20.1 pg)	MCHC[15] (36-41 g/dL)	RDW[16] (17-21%)	Platelets (135-650 x103/μL)	MPV[17] (4.7-8.0 fL)
27	8.2	500	43	16	37	17	376	6.3
27	7.7	400	44	16	38	17	329	6.7
28	8.0	500	44	16	37	17	154	7.6
26	8.1	500	42	15	36	20	299	6.1
26	8.0	400	42	15	36	20	239	7
24	7.9	400	42	16	37	20	213	6.6
29	7.8	300	41	15	37	17	131	8.3
29	8.0	400	40	15	36	17	380	6.2
29	7.5	200	40	15	37	17	386	6.8
30	7.6	400	44	16	36	18	435	6.7
28	7.5	400	43	16	37	18	448	7.1
30	7.6	300	44	16	37	18	421	7.6
30	8.5	400	44	16	36	16	387	5.9
29	8.5	600	44	16	36	16	318	6.7
28	8.2	400	44	16	36	16	354	6.1
29	7.7	100	41	14	34	19	352	6.9
31	7.8	300	41	14	35	20	234	6.7
30	8.0	500	41	14	36	19	340	7.3
31	8.0	500	49	17	35	17	378	6.6
31	8.1	500	50	17	35	17	365	6.8
34	8.7	400	49	18	36	17	383	6.8
28	7.0	500	44	16	36	17	335	5.9
29	7.2	300	44	16	36	16	542	5.9
27	7.2	400	43	16	36	17	480	6.4
24	7.5	1000	45	16	37	17	373	6.6
23	7.9	800	44	16	37	18	479	7.1
24	7.7	600	45	16	36	18	530	7.2
30	8.1	500	43	15	35	18	292	5.7
31	8.5	500	44	16	36	18	386	6.4
30	8.2	600	43	15	35	17	381	6.2
28	7.6	500	40	14	36	19	303	6.6

26	7.4	600	40	14	36	20	424	6.1
29	7.8	300	41	15	36	21	371	6.3
28	8.3	800	44	16	36	17	244	6.3
27	8.5	700	45	16	36	18	488	6.9
27	8.3	700	45	16	36	19	407	6.8
30	8.7	700	46	17	37	17	512	6.2
30	8.5	500	46	16	36	16	576	6.1
29	8.4	700	46	17	36	16	542	6.2
24	8.1	900	45	16	34	17	387	7.5
25	8.0	600	44	16	35	18	494	6.9
27	7.4	400	46	16	34	20	502	7.3
23	8.4	900	40	15	37	18	219	11.5
26	8.6	600	41	15	36	19	176	11.9
22	8.3	600	40	15	38	19	88	11.2
30	7.7	700	46	17	37	17	388	6.7
30	8.1	400	46	17	37	17	478	6.6
32	8.2	400	46	17	37	17	407	6.7
26	8.0	1100	46	16	36	17	146	9
26	8.6	800	46	16	36	17	208	8.4
25	8.6	800	46	16	35	18	184	7.9
30	9.5	1000	41	15	36	19	599	6.9
28	8.6	800	41	16	38	19	436	6.8
28	8.6	700	41	15	37	20	471	7.1
29	7.8	800	43	16	37	17	766	6.7
27	7.6	600	43	15	35	17	811	6.3
26	7.3	500	43	15	35	18	601	7.2
32	8.8	600	49	18	37	15	479	5.6
28	8.8	700	50	18	36	16	442	6.4
28	8.4	400	49	18	37	17	352	6.5

[1]WBC = white blood cells
[2]Band# = band cells
[3]Seg# = segmented cells
[4]Lymph# = lymphocytes

[5]Mono# = monocytes
[6]Eosin# = eosinophils
[7]Baso# = basophils
[8]Meta# = metamyelocytes
[9]RBC = red blood cells
[10]Hgb = hemoglobin
[11]PCV = packed cell volume
[12]Pprot = plasma protein
[13]MCV = mean corpuscular volume
[14]MCH = mean corpuscular hemoglobin
[15]MCHC = mean corpuscular hemoglobin concentration
[16]RDW = red cell distribution width
[17]MPV = mean platelet volume
