# Supplementary material for: A Fixed Cohort Field Study of Gene Expression in Circulating Leukocytes From Dairy Cows With and Without Mastitis
Source: Front Vet Sci. 2020 Sep 30;7:559279. doi: 10.3389/fvets.2020.559279 (PMC7554338; doi:10.3389/fvets.2020.559279)
Supplement: Supplementary file 2 [file Data_Sheet_2.pdf]

Table S2. Serum biochemical data for cows with and without mastitis at all sampling points. Reference cut-points are provided within parentheses, and cells with grey coloration indicate values outside established reference ranges.

Cow ID	Mastitis (Y/N)	Sampling Dates	SDH[1] (0-24 U/L)	GGT[2] (12-80 U/L)	AST[3] (50-320 U/L)	ALP[4] (13-183 U/L)	CK[5] (30-360 U/L)	BUN[6] (7-19 mg/dL)	Creatinine (0.8-1.4 mg/dL)	Glucose (40-80 mg/dL)	TP[7] (5.8-8.3 g/dL)	Albumin (2.9-4.1 g/dL)	Globulin (2.5-4.9 g/dL)	Calcium (8.4-10.8 mg/dL)
3731	N	2/18/19	16	35	81	47	234	15	0.6	61	7.9	3.2	4.7	9.4
		2/25/19	12	34	69	48	148	14	0.6	65	7.7	3.1	4.6	8.6
		3/5/19	15	37	80	49	183	16	0.6	69	8.1	3.3	4.8	9.9
5307	N	2/7/19	12	41	80	47	283	12	0.7	52	7.8	3.3	4.5	9
		2/14/19	10	38	82	53	154	22	0.7	68	8	3.3	4.7	9.7
		2/21/19	15	37	85	52	352	8	0.8	58	7.7	3.2	4.5	10.2
5362	N	3/5/19	10	47	108	68	815	18	0.7	73	7.9	3.6	4.3	9.9
		3/11/19	14	45	82	65	148	25	0.8	71	7.7	3.5	4.2	9.7
		3/19/19	16	46	83	65	122	21	0.7	64	7.7	3.4	4.3	9.4
6081	N	3/5/19	18	48	100	95	189	13	0.5	73	7.5	3.4	4.1	10
		3/11/19	19	48	78	90	183	13	0.5	69	7.2	3.3	3.9	9.5
		3/19/19	20	48	82	87	169	16	0.5	62	7.4	3.3	4.1	10
6246	N	2/18/19	12	48	75	51	184	14	0.5	65	8.2	3.4	4.8	9.4
		2/25/19	8	48	66	50	155	17	0.5	72	8.3	3.3	5	9
		3/5/19	11	45	77	59	176	14	0.5	67	8.2	3.4	4.8	9.9
6748	N	3/11/19	13	35	75	58	157	23	0.6	63	7.9	3.1	4.8	8.9
		3/19/19	16	33	83	54	195	18	0.6	64	7.7	3.1	4.6	9.3
		3/26/19	14	31	81	41	214	9	0.4	65	8.1	3.1	5	10.5
8494	N	3/19/19	21	34	87	144	164	12	0.7	57	7.6	3.5	4.1	9.7
		3/26/19	22	35	94	138	171	12	0.7	61	7.8	3.5	4.3	8.6
		4/2/19	26	38	99	153	185	13	0.7	64	8.1	3.6	4.5	9.7
11150	N	3/5/19	17	30	93	76	265	19	0.5	62	6.7	3.1	3.6	9.2
		3/11/19	17	32	100	94	806	19	0.6	61	7	3.2	3.8	9.7
		3/19/19	27	34	122	90	554	16	0.6	64	7	3.1	3.9	9.6
3955	Y	3/11/19	12	29	99	57	105	18	0.6	77	6.6	1.8	4.8	7.8
		3/19/19	15	26	95	34	256	10	0.5	67	7.7	1.8	5.9	8.4
		3/26/19	24	29	95	39	117	10	0.6	66	7.5	2	5.5	9.5
4451	Y	2/7/19	5	35	65	49	102	13	0.7	84	7.8	3.4	4.4	9.2

		2/14/19	5	34	82	52	612	12	0.6	66	8.4	3.4	5	9
		2/21/19	6	34	71	47	120	7	0.9	63	8	3.2	4.8	9.8
		2/14/19	10	44	82	105	134	14	0.6	74	7.4	3.3	4.1	9.7
5291	Y	2/21/19	9	39	78	92	221	10	0.6	60	7	3.1	3.9	8.9
		3/1/19	11	40	106	93	597	15	0.7	63	7.2	3.2	4	10
		2/18/19	5	39	61	42	87	12	0.5	80	7.5	3.1	4.4	8.6
5410	Y	2/25/19	24	43	109	57	122	16	0.4	69	8.3	2.9	5.4	9.3
		3/5/19	19	50	103	66	139	16	0.5	72	8.4	3.1	5.3	9.4
		2/25/19	10	40	69	80	113	18	0.5	69	8.4	3.3	5.1	8.9
5754	Y	3/5/19	12	42	84	96	182	21	0.5	61	8.4	3.4	5	10.2
		3/11/19	15	42	78	112	126	17	0.5	64	8.4	3.2	5.2	9.7
		3/11/19	5	42	63	172	80	12	0.6	74	7.5	2.9	4.6	8.4
6341	Y	3/19/19	10	39	83	263	99	18	0.6	63	7.9	2.9	5	9
		3/26/19	1	39	94	368	189	9	0.7	66	7.9	3.1	4.8	10.3
		2/25/19	9	39	82	60	346	16	0.6	68	7.9	2.8	5.1	9
6493	Y	3/5/19	9	41	102	87	422	14	0.7	66	8.6	3	5.6	10.2
		3/11/19	14	39	83	76	151	18	0.7	68	8.1	2.8	5.3	9.1
		2/14/19	26	40	97	55	198	11	0.4	77	7.5	3.2	4.3	8.2
6645	Y	2/21/19	12	42	71	72	157	9	0.5	61	8	3.3	4.7	8.8
		3/1/19	10	38	71	71	170	17	0.5	71	8	3.3	4.7	10.8
		2/14/19	7	50	61	101	115	12	0.6	72	7.2	2.9	4.3	7.6
6805	Y	2/21/19	10	49	74	116	129	5	0.6	54	8.3	2.8	5.5	7.9
		3/1/19	11	52	80	150	199	12	0.5	64	8.1	2.9	5.2	9.5
		3/19/19	5	33	64	38	114	11	0.5	74	8.8	2.9	5.9	10.3
9028	Y	3/26/19	13	31	74	34	137	7	0.5	70	8.4	2.8	5.6	10.1
		4/2/19	15	32	68	41	117	14	0.6	69	8.3	2.9	5.4	10.1
		2/25/19	28	119	77	102	157	17	0.5	73	7	3.1	3.9	8.6
11151	Y	3/5/19	6	86	75	117	132	14	0.5	68	7.4	3.2	4.2	9.7
		3/11/19	11	72	70	129	136	17	0.5	67	7	3.2	3.8	9.6
		2/7/19	12	26	89	46	176	15	0.8	69	8.8	3	5.8	9
20008	Y	2/14/19	11	25	85	58	121	12	0.5	70	8.8	2.7	6.1	9.4
		2/21/19	10	24	77	59	109	8	0.6	67	8.6	2.8	5.8	9.1

7

Phosphorus (3.6-8.1 mg/dL)	Magnesium (1.6-3.0 mg/dL)	Sodium (133-148 mEq/L)	Potassium (3.7-5.8 mEq/L)	Chloride (94-109 mEq/L)	CO2 (18-34 mEq/L)	AnionGap (12-28 mEq/L)
5.1	2.2	142	4.2	101	26.5	18.7
4.9	2.9	141	5.3	101	26.8	18.5
5.1	2.1	140	4.4	99	23.6	21.8
6.6	2.1	142	4.3	103	23	20.3
7.1	2.3	141	4.2	99	25.8	20.4
5.7	2	139	4.8	101	23.8	19
6.2	2.5	140	5.2	100	22.3	22.9
5.9	2.2	142	4.7	98	29.3	19.4
7	2.5	139	4.6	96	31.2	16.4
5.7	2	143	4.8	101	23.4	23.4
6.2	2	141	4.3	98	26.4	20.9
6.8	2	141	4.5	98	27.6	19.9
5	2.6	139	4.5	99	23.6	20.9
4.5	2.4	143	4.2	101	26	20.2
5	2.4	140	4.8	100	23.3	21.5
6.4	3	145	4.5	102	25.9	21.6
5.9	2.7	143	4.9	99	29.8	19.7
7.1	2.2	141	4.5	100	25.9	19.6
3.4	2.7	141	5.1	97	30.2	18.9
3.5	3.4	142	5.1	102	25.6	19.5
2.9	3.1	143	5.2	101	29.2	18
6.1	2.4	139	4.3	99	25	19.3
5.2	2.7	142	4.5	99	27.4	20.1
5.1	2.5	143	4.1	101	28.4	17.7
4.5	2.6	142	3.9	102	27.7	16.2
4.6	2.1	137	4.3	100	26.3	15
6.8	2.1	139	4.9	101	28.6	14.3
6.7	2.2	143	5.5	105	23.7	19.8
6.1	2.4	141	5.3	103	21.5	21.8
5.8	2.3	141	5.1	103	23.8	19.3
6.8	2.1	142	4.9	102	26.8	18.1

6.5	3	141	4.5	103	24.7	17.8
6.7	2.1	141	4.8	100	29.4	16.4
5.5	2.4	140	4.2	102	23.6	18.6
5.5	2.7	140	4.6	100	25.8	18.8
5.8	2.5	138	4.6	98	22.7	21.9
4.6	2.5	141	4.1	99	26.7	19.4
4.7	2.5	142	4.7	100	24.7	22
4.8	2.2	141	5	100	26.1	19.9
3.4	2.4	143	4.5	103	24.9	19.6
4.1	2.7	143	3.8	101	27.7	18.1
6	2.5	142	4.8	102	28.3	16.5
6.7	3.2	140	4.8	102	25.2	17.6
6.4	2.4	142	5.2	102	20.8	24.4
7.7	2.5	143	4.1	101	24.1	22
3.6	2.1	142	3.8	100	27	18.8
5.4	2.4	141	4.3	100	26.1	19.2
7.5	1.9	141	4.2	98	30.1	17.1
4.1	1.8	145	3.8	102	28.7	18.1
3.2	2	141	4.2	100	28.1	17.1
4.9	2.5	135	5	97	27.1	15.9
4.6	2.3	141	4.9	99	27	19.9
6.6	2.3	140	4.3	100	25.4	18.9
7.8	2.2	144	4	100	30.8	17.2
5.9	3.1	139	5.3	97	30.6	16.7
5.9	2.8	144	4.8	100	25.1	23.7
5.7	2.7	144	4.7	100	29.2	19.5
6.1	2	140	4.6	103	24.7	16.9
5.1	2.1	142	4.2	101	28.4	16.8
5.5	2.1	140	4.3	102	24.6	17.7

[1]SDH = sorbitol dehydrogenase
[2]GGT = gamma-glutamyl transferase
[3]AST = aspartate aminotransferase
[4]ALP = alkaline phosphatase
[5]CK = creatine kinase

[6]BUN = blood urea nitrogen

[7]TP= total protein
